# Chemical profiling, antioxidant, and antibacterial properties of *Tropaeolum majus* L. extract as a functional food ingredient

**DOI:** 10.3389/fnut.2025.1626562

**Published:** 2025-09-24

**Authors:** Eliana-Yissel Aguilera-Angel, Diego Ballesteros-Vivas, Ricardo Vera-Bravo, Néstor García, Jorge-Eliecer Robles-Camargo, Geison Modesti Costa, Mauricio Espinal-Ruiz, Juan Pablo Caicedo-Trejos, Ana Karina Carrascal Camacho, Izlia-Jazheel Arroyo-Maya, Elena Ibáñez, Alejandro Cifuentes, Valentina Guzmán-Pérez

**Affiliations:** 1 Departamento de Nutrición y Bioquímica, Facultad de Ciencias - Pontificia Universidad Javeriana, Bogotá, Colombia; 2 Escuela de Ciencias Básicas Tecnología e Ingeniería, Grupo DAVINCI, Universidad Nacional Abierta y a Distancia, Bogotá, Colombia; 3 Departamento de Química, Facultad de Ciencias - Pontificia Universidad Javeriana, Bogotá, Colombia; 4 Departamento de Biología, Facultad de Ciencias - Pontificia Universidad Javeriana, Bogotá, Colombia; 5 Center for Research in Energy and Environment (CREE), University of Missouri of Science and Technology, Rolla, MO, United States; 6 Laboratorio de Microbiología de Alimentos, Grupo de Biotecnología Ambiental e Industrial, Departamento de Microbiología, Facultad de Ciencias - Pontificia Universidad Javeriana, Bogotá, Colombia; 7 Departamento de Procesos y Tecnología - División de Ciencias Naturales e Ingeniería, Universidad Autónoma Metropolitana – Unidad Cuajimalpa, Ciudad de México, Mexico; 8 Foodomics Laboratory, Institute of Food Science Research (CIAL) (CSIC-UAM), Madrid, Spain

**Keywords:** nasturtium, *Tropaeolum majus*, benzyl glucosinolate, antioxidant capacity, antibacterial activity

## Abstract

**Background:**

Nasturtium (*Tropaeolum majus* L.) is an edible plant that contains a diverse array of bioactive compounds, including phenolics, glucosinolates, and their hydrolysis products, isothiocyanates. Despite its functional potential, the plant remains largely underutilized, as it is predominantly cultivated as an ornamental species. Its limited incorporation into food products is primarily attributed to the sulfurous odor and pungent, bitter taste generated by glucosinolate degradation products. Concentrating these bioactive compounds through extraction offers a promising approach to reduce the quantity of plant material needed for functional enrichment.

**Methods:**

The aim of this study was to identify the bioactive compounds and functional potential of nasturtium leaves and flowers collected in Cogua, Cundinamarca (Colombia). For this purpose, fresh samples were harvested, processed, and subjected to a preliminary phytochemical analysis. Methanolic and ethanolic extracts were prepared, and total glucosinolates, phenolics, and flavonoids were quantified. Chemical profiling was performed using UHPLC-q-TOF-MS/MS, while functional properties were assessed through antioxidant and antibacterial assays.

**Results:**

The ethanolic extract (70% v/v ethanol/water) from leaves retained phenolic compounds (2.10 ± 0.19 g GAE/g DS) and flavonoids (0.15 ± 0.02 g QE/g DS) which are linked to antioxidant activity, and benzyl glucosinolate (8.47 ± 1.68 μmol SE/g DW), whose hydrolysis product, benzyl isothiocyanate, is associated with antibacterial effects. Consistent with this, Enterococcus faecalis (Gram-positive) showed the lowest minimum inhibitory concentration (MIC, 15.6 mg/mL), while *Escherichia coli* and *Salmonella Typhimurium* (Gram-negative) exhibited MICs of 31.3 mg/mL.

**Conclusion:**

This study provides the first report of glucosinolate content in nasturtium cultivated in Colombia and describes the preparation and characterization of an extract obtained with generally recognized as safe (GRAS) solvents. The findings highlight its potential application in functional foods and nutraceuticals.

## 1 Introduction

Nasturtium (*Tropaeolum majus* L.) is a plant native to the Andes of South America and is commonly used as an ornamental plant (1). However, its leaves, flowers, and unripe green seeds are edible and are often used to add a spicy flavor to salads and sauces (2–8). Nasturtium is widely recognized for its high functional potential attributed to its rich content of bioactive compounds, such as phenolic compounds (PC), Glucosinolates (GLSs), and their hydrolysis products, the isothiocyanates (ITCs) (9–11).

GLSs are secondary plant metabolites, and their backbone chemical structure includes a *β*-D-glucopyranose residue linked to thiohydroximate-O-sulfonate (12) and a variable R group derived from amino acids. Based on the amino acid-derived R group, GLSs are classified as aliphatic, aromatic, or indole (12, 13). GLSs are stored in the plant and hydrolyzed by the enzyme myrosinase when the plant is exposed to damage, microbial attack, or other stress conditions. This reaction produces an unstable aglycone, which subsequently forms different compounds depending on the nature of the R group and various physicochemical factors, such as pH, the presence of specific enzymes, and ferrous ions (Fe^2+^) (12). Under neutral pH conditions, the hydrolysis of benzyl glucosinolate in nasturtium leads to the formation of benzyl isothiocyanate (BITC), a compound highly susceptible to degradation due to temperature shifts and extended processing (14).

The quantification of GLSs can be carried out using different methodologies. The most used is high-performance liquid chromatography coupled with a diode array detector (HPLC-DAD). Regarding GLSs extraction, methodologies involving the derivatization of GLSs, such as the formation of desulfoglucosinolates using sulfatase, can be employed. However, this method requires extended processing times and the use of expensive reagents. In contrast, the extraction of intact GLSs prevents the degradation of the compounds, thereby ensuring a more efficient and cost-effective process (15).

Currently, there are no reports on the GLSs content in nasturtium flowers and leaves from Colombia. Therefore, developing a rapid method for determining GLSs content in nasturtium could facilitate further research on this plant. In addition to GLSs, Colombian nasturtium flowers have been reported to contain high levels of PC, including anthocyanins, cinnamic acid, quinic acid derivatives, and flavonoids such as myricetin, quercetin, and kaempferol derivatives (11, 16–18). The GLSs and PC found in nasturtium may be linked to the health-promoting effects reported in previous studies (19). *In vivo* and *in vitro* studies have demonstrated that nasturtium consumption can influence the secretion of neuropeptides involved in energy balance, regulate lipoprotein metabolism, and modulate anti-inflammatory and antioxidant biomarkers (20–23).

The presence of bioactive molecules, such as PC in nasturtium, contributes to its antioxidant activity according to earlier studies for this plant (11, 16, 24, 25). The antioxidant capacity of nasturtium has been measured using complementary methodologies, including DPPH (free radical scavenging activity), ABTS (radical scavenging activity), FRAP (ferric reducing antioxidant power), CUPRAC (cupric ion (Cu^2+^) reducing capacity), and ORAC (oxygen radical absorbance capacity) (11, 16, 24, 25). Colombian nasturtium flowers exhibit high antioxidant capacity, as measured by ORAC (16). However, antioxidant activity in Colombian leaves, as well as data from other complementary methods have not yet been reported.

The antibacterial activity of the extract was studied to evaluate its potential use as a natural food preservative. In addition to its antioxidant activity attributed to its phenolic compound content, nasturtium also exhibits antimicrobial properties. The main antimicrobial compound is benzyl isothiocyanate (BITC), a hydrolysis product of benzyl glucosinolates (26). BITC has demonstrated strong activity against various bacterial pathogens, such as *Escherichia coli* and *Salmonella Typhimurium* (27). Therefore, in addition to the functional properties that nasturtium extract can offer as a bioactive ingredient, it could also contribute to microbial protection, supporting its potential application in food preservation.

Some reports describe the development of foods enriched with freeze-dried nasturtium or its extracts, primarily in baked goods and beverages. For example, Krell et al. (14, 28) formulated breads enriched with 2.5 and 4% freeze-dried nasturtium leaves, respectively. Platz et al. (29) and Schiess et al. (23) prepared aqueous suspensions using 10 grams of freeze-dried leaves dissolved in 50 mL of water. These studies focus on evaluating the bioavailability, stability, and functional properties of the bioactive compounds present in nasturtium. However, it has been reported to impart a characteristic pungent and bitter taste and odor to food, commonly associated with glucosinolate breakdown products (2, 20, 30). To address this issue, one strategy is to concentrate its bioactive compounds through extract production, thereby reducing the amount of plant material needed for enrichment. Current research on the food use of nasturtium and its extracts has focused on encapsulation and delivery strategies aimed at masking undesirable sensory attributes while preserving their bioactive properties. Therefore, the development and characterization of a nasturtium extract to concentrate its bioactive compounds could facilitate its incorporation into foods or nutraceuticals, preserving its health benefits while minimizing sensory alterations.

Due to that the content of bioactive compounds in plants can vary according to environmental factors specific to their origin, as well as differences in extraction methodologies, this study aims to investigate the chemical composition and functional potential of nasturtium leaf and flower collected in Cogua, Cundinamarca (Colombia).

It is hypothesized that the phytochemical profile of leaf and flower extracts of *Tropaeolum majus* grown in Colombia differs from that reported in other regions of the world, particularly in the content of glucosinolates and flavonoids. These variations, influenced by edaphoclimatic conditions and plant tissue type, are expected to modulate the bioactivity of the extracts and their associated functional potential.

This study advances in the phytochemical characterization and functional potential identification of nasturtium, and represents the first report of GLSs in nasturtium plants in Colombia, This study made a progressing the characterization of an extract obtained using generally recognized as safe (GRAS) solvents and an extraction methodology designed to preserve bioactive compounds particularly glucosinolates, which are highly susceptible to degradation during conventional extraction processes. All together represents an opportunity to promote the potential use of this underutilized plant as a functional ingredient in the food, nutraceutical, and cosmetic industries.

## 2 Materials and methods

### 2.1 Chemicals and reagents

Sinigrin was supplied as a potassium salt with a purity of 99.83% w/w by PhytoLab GmbH & Co. KG (Vestenbergsgreuth, Germany). Myrosinase (thioglucosidase from *Sinapsis alba*—white mustard seed) with an enzymatic activity of 187.4 U/g was purchased from Sigma-Aldrich Corp. (St. Louis, MO, United States). DPPH (2,2-diphenyl-1-picrylhydrazyl) and Trolox (6-hydroxy-2,5,7,8-tetramethylchroman-2-carboxylic acid) were obtained from Merck (Darmstadt, Germany). Methanol, acetone, fluorescein sodium (FL), phosphate buffer (PBS) prepared from dipotassium hydrogen phosphate (K₂HPO₄) and potassium dihydrogen phosphate (KH₂PO₄), 2,2′-azobis(2-amidinopropane) dihydrochloride (AAPH), 6-hydroxy-2,5,7,8-tetramethylchroman-2-carboxylic acid (Trolox), potassium persulfate (K₂S₂O₈), and neocuproine (C₁₄H₁₂N₂) were obtained from Sigma-Aldrich (Madrid, Spain). Acetonitrile (C₂H₃N) was purchased from J. T. Baker (Madrid, Spain), and diammonium 2,2′-azino-bis(3-ethylbenzothiazoline-6-sulfonate) (ABTS) was obtained from BIOBASIC (Markham, ON, Canada). Ultrapure water was produced using a Millipore system (Billerica, MA, United States). All other chemicals were purchased from PanReac AppliChem (Barcelona, Spain) and J. T. Baker (Phillipsburg, NJ, United States).

### 2.2 Plant material

Nasturtium plants were grown in a greenhouse at the Pontificia Universidad Javeriana, located in Cogua, Cundinamarca, Colombia (5°04′09 ″ N, 73°52′48″ W, and 2,580 MASL), the cultivated seeds were sourced from wild-growing specimens native to the same region. During cultivation, the greenhouse temperature ranged between 6.5 and 32°C. The plant material was collected during the flowering stage, avoiding open flowers, flower buds, and senescent flowers. After harvesting, the leaves and flowers were immediately frozen, freeze-dried, and milled into fine powder. The powder of leaves and flowers was stored individually in a dark environment at −20°C until use.

### 2.3 Preliminary phytochemical characterization of nasturtium leaves and flowers

For this assay, nine different extracts were obtained through solid–liquid extraction from freeze-dried nasturtium leaves and flowers.

Benzine and dichloromethane were used to remove low-polarity compounds that could act as interferents. Subsequently, ethyl acetate and ethanol were used as solvents for the preliminary phytochemical characterization (31). A solid-to-liquid ratio of 1:15 was used, and the mixtures were sonicated for 10 min at room temperature. The first extractions from lyophilized flowers and leaves (F and L) were performed with (B) benzine as the solvent (F-B and L-B, respectively); the resulting solid residue from flowers and leaves were then used for subsequent extractions with (D) dichloromethane (F-D and L-D, respectively), (A) ethyl acetate (F-A and L-A, respectively), and finally (E) ethanol (F-E and L-E, respectively). Additionally, a hydroethanolic extract from nasturtium leaves, prepared as described in Section 2.4.1.2., was included in the analysis.

The extracts mentioned before were analyzed by high-performance thin-layer chromatography (HPTLC), using silica gel as the stationary phase. A sample volume of 3 μL and a standard volume of 2 μL were applied. The specific chromatographic systems employed for the metabolite groups previously reported in nasturtium (16, 24) are summarized in Table 1.

**Table 1:** Chromatographic conditions (HPTLC) used in the preliminary analysis of different extracts from nasturtium leaves and flowers.

Metabolite	Mobile phase	Proportion (mL)	Developer/visualization	Standards (1 mg/mL)
Flavonoids—glycosides	Ethyl acetate: Formic acid: Acetic acid: Water	100:11:11:26 (v/v/v/v)	Natural reagent/UV 366 nm	Quercetin and Rutin
Flavonoids—Aglycones	n-Hexane: Ethyl acetate: Formic acid	10:6:1 (v/v/v)	Natural reagent/UV 366 nm	Quercetin and Rutin
Phenolic acids	Toluene: Ethyl acetate: Formic acid	9:4:1 (v/v/v)	Natural reagent/UV 366 nm	Caffeic and Chlorogenic acids

### 2.4 Characterization of bioactive compounds and antioxidant capacity in methanolic and ethanolic extracts of nasturtium

#### 2.4.1 Extraction procedures

##### 2.4.1.1 Methanolic extract as reference

The preliminary phytochemical characterization of nasturtium leaves and flowers showed that most of the detected compounds were phenolic and associated with polar solvents.

Consequently, the analysis focused on methanolic extracts from the leaves (ML) and flowers (MF) as reference samples. Freeze-dried plant material (20 mg) was weighed into a test tube, and 850 μL of 70% methanol/water was added. The extraction was performed in three successive steps: the samples were initially heated at 80°C for 10 min (with evaporated solvent replenished every 2 min), followed by centrifugation at 16,000 × g for 10 min. The resulting supernatants were collected in a test tube, lyophilized, and stored at −20°C for further use.

##### 2.4.1.2 Ethanolic extract for food applications

To obtain an extract suitable for use as a food ingredient, nasturtium leaves were selected for the preparation of an ethanolic extract. Leaves were chosen over flowers due to their greater availability in cultivation, ensuring a sustainable raw material supply for future trials.

The ethanolic extract from leaves (ELE) was prepared following a modified version of a method described previously (32). Five grams of freeze-dried leaves were mixed with 75 mL of 70% ethanol/water and heated at 75°C for 5 min under constant stirring, followed by rapid cooling in an ice bath. The extract was subjected to ultrasonic treatment for 15 min, filtered, and concentrated to 15 mL using a rotary evaporator set at 40°C and 75 mBar. Finally, the extract was lyophilized and stored at −20°C until further use.

#### 2.4.2 Chromatographic analysis of intact GLSs

##### 2.4.2.1 Sample preparation

MF, ML, and ELE were reconstituted in a solution containing 150 μL of 70% v/v methanol/water, 200 μL of 0.4 M barium acetate, and 650 μL of ultra-pure (MilliQ) water. The extracts were incubated at room temperature for 30 min, followed by centrifugation at 16,000 × g for 10 min. The supernatants were collected, and the final volume was adjusted to 2 mL with ultra-pure water.

The extracts were aliquoted into two tubes, each containing 1 mL of the mixture. In the first tube, 7 μL of ultra-pure water was added as a control, while in the second tube, 7 μL of myrosinase (0.01 U/μL) was added. The samples were incubated at 37°C for 8 h, followed by filtration through a 0.22 μm PVDF membrane. The filtrates were then transferred to vials and stored at −80°C until further analysis.

##### 2.4.2.2 GLSs quantification method

The determination of intact GLSs in MF, ML, and ELE was performed according to the method proposed by Förster et al. (15) with some modifications. A Shimadzu Prominence 20 series chromatograph coupled to a diode array detector set at 229 nm was used for qualitative and quantitative analysis. For this, 10 μL of MF, ML or ELE was injected into a Reprosil Star SB-C18 column (5 μm, 250 mm × 4.6 mm, Dr. Maisch brand) operated at 37°C and eluted using a solvent system consisting of solvent A (100% 0.1 M ammonium acetate) and solvent B (40% acetonitrile/0.1 M ammonium acetate). The gradient program used was as follows: 0–2 min: 0–1% B; 2–20 min: 1–50% B; 20–24 min: 50–100% B; 24–26 min: 100% B; 26–27 min: 100–1% B; and 27–35 min: 1–0% B, at a flow rate of 1.5 mL/min. Quantification was carried out using a sinigrin (15) calibration curve with 12 points, covering a concentration range between 0.5 and 500 μM (*n* = 3). GLSs concentrations were determined by interpolation on this calibration curve.

Results were expressed as μmol of sinigrin equivalents per gram of dry sample (μmol EE/g DS). Sinigrin, also known as allyl glucosinolate, was used as the standard due to its structural similarity (1-S-[(1Z)-N-(sulfonatooxy) but-3-enimidoyl]-1-thio-beta-D-glucopyranose) to benzyl glucosinolate (1-S-[(1Z)-2-phenyl-N-(sulfonatooxy) ethanimidoyl]-1-thio-beta-D-glucopyranose) and its thermostability.

The chromatographic method was standardized according to the parameters proposed by Magnusson et al. (33) as described below. The limit of detection (LOD) and limit of quantification (LOQ) were determined based on repeated measurements of sinigrin at low concentrations (*n* = 10), using the standard deviation of replicate measurements near the LOD multiplied by an appropriate factor, as recommended to ensure reliable and representative estimates. Linearity within the working concentration range was evaluated using a 12-point calibration curve, with each point measured in experimental triplicate. Method accuracy was assessed through percent recovery using a glucosinolate standard (sinigrin) as a reference. The impact of the extraction procedure on sinigrin recovery was evaluated by comparing the chromatographic signal of extracted versus non-extracted samples. Precision was determined by quantifying 10 experimental replicates known as sinigrin concentrations, as well as 10 technical replicates of the same concentration (33).

#### 2.4.3 Total phenolic content

The dry extracts (MF, ML, and ELE) were dissolved in methanol (2 mg/mL), and 20 μL of this solution were mixed with 1,580 μL of distilled water and 100 μL of Folin–Ciocalteu reagent. The mixture was stirred and incubated for 8 min, followed by the addition of 300 μL of a 20% sodium carbonate solution. It was incubated in the dark at room temperature for 2 h (34). Absorbance was measured at 765 nm using an automated plate reader (Cytation 5, Bio Tek, United States).

The calibration curve was prepared with a gallic acid solution in methanol (*n* = 3), with concentrations ranging from 0.01 to 1.4 mg/mL. Results were expressed as grams of gallic acid equivalent per gram of dry extract (g GAE /g DS), based on the calibration curve of gallic acid.

#### 2.4.4 Total flavonoids content

The dry extracts (MF, ML, and ELE) were dissolved in methanol (2 mg/mL), and 100 μL of this solution was mixed with 300 μL of ethanol (95%), 20 μL of aluminum chloride (10%), 20 μL of potassium acetate (1 M), and 560 μL of distilled water. The mixture was stirred and incubated in the dark at room temperature for 40 min (34). Absorbance was measured at 415 nm using an automated plate reader (Cytation 5, Bio Tek, United States). A calibration curve was prepared using a quercetin solution in methanol (*n* = 3), with concentrations ranging from 0.02 to 0.2 mg/mL. Results were expressed as mg quercetin equivalent per gram of dry extract (g QE/g DS), based on the quercetin calibration curve.

#### 2.4.5 Determination of antioxidant capacity

The antioxidant capacity of MF, ML, and ELE extracts was assessed using different methodologies, including DPPH (radical scavenging capacity), ABTS [2,2′-azino-bis (3-ethylbenzothiazoline-6-sulfonic acid)], and CUPRAC (cupric ion reducing antioxidant capacity) (35–37). The results were expressed as μmol of Trolox equivalents per gram of DS (μmol TE/g DS).

##### 2.4.5.1 ABTS assay

The radical scavenging activity was determined using the ABTS assay. A 40 mL aqueous solution of ABTS (7 mM) was mixed with 704 μL of potassium persulfate (140 mM) to obtain a final concentration of 2.45 mM. The solution was incubated in the dark at room temperature for 12–24 h and subsequently diluted with 0.075 M PBS (pH 7.0) to achieve an absorbance of 734 nm. Then, 1,450 μL of the ABTS cationic radical solution was combined with 25 μL of ML, MF or ELE (0.05 mg/mL) and 25 μL of PBS. The mixture was stirred for 30 s, after which 250 μL was transferred to a plate. Absorbance at 734 nm and 30°C was recorded at 0 and 60 min after radical neutralization. Quantification was performed using a Trolox calibration curve ranging from 10 to 50 μM (37).

##### 2.4.5.2 CUPRAC assay

The cupric ion reducing antioxidant capacity (CUPRAC) assay was analyzed using an automated plate reader (Cytation 5, Bio Tek, United States) (37). The CUPRAC reagent was prepared by mixing 62 μL of copper chloride solution (10 mM in water), 62 μL of Neocoproine solution (7.5 mM, in ethanol), and 62 μL of acetate buffer (1.0 M, pH 7.0). Then, 64 μL of MF, ML (0.5 mg/mL), or ELE (1 mg/mL) were added. The mixture was stirred for 30 s and incubated at room temperature in the dark for 60 min. Absorbance was measured at 450 nm and 25°C. The quantification was performed using a Trolox calibration curve ranging from 12 to 200 μM.

##### 2.4.5.3 DPPH assay

Antiradical efficiency was determined using the DPPH method. For this assay, 100 μL of MF or ML extract (2 mg/mL) or ELE (1 mg/mL) diluted in methanol was mixed with 900 μL of DPPH reagent (40 μg/mL methanol) and incubated in the dark for 30 min. Absorbance was measured at 517 nm using a Thermo SCIENTIFIC EVOLUTION 201 spectrophotometer. Quantification was performed using a Trolox calibration curve ranging from 6 to 35 μM.

### 2.5 Ultra-high performance liquid chromatography coupled to quadrupole time-of-flight tandem mass spectrometry (UHPLC-QTOF-MS/MS) analysis of ethanolic extract of nasturtium leaves

Given that ELE was produced for food applications, a more detailed characterization of its composition was required. The analysis proceeded with the tentative identification of the compounds present in the extract using liquid chromatography coupled with high-resolution mass spectrometry (LC-HRMS). For this purpose, an ultra-high-performance liquid chromatography-quadrupole time-of-flight tandem mass spectrometry (UHPLC-q-TOF-MS/MS) analysis was performed.

Chromatographic separation was conducted using an Agilent 1,290 UHPLC system (Agilent Technologies, Santa Clara, CA), with a reversed-phase column (Zorbax Eclipse Plus C18, 2.1 × 100 mm, 1.8 μm particle diameter, Agilent Technologies, Santa Clara, CA) at 30°C. A 5.0 μL aliquot of the ELE was injected. Solvent A was a formic acid solution (0.01% v/v), and solvent B was ACN. Solvents were delivered at a flow rate of 0.5 mL/min during gradient elution as follows: 0 min, 0% B; 7 min, 30% B; 9 min, 80% B; 11 min, 100% B; 13 min, 100% B; 14 min, 0% B. The chromatographic system was interfaced with an Agilent 6,540 quadrupole time-of-flight mass spectrometer (qTOF MS) through an orthogonal ESI source. The system was operated in the negative-ion mode, and the source and mass spectrometric parameters were optimized as follows: ion spray capillary voltage, 4,000 V; nebulizer pressure, 40 psi; nebulizer gas flow rate, 10 L/min; gas temperature, 350°C; skimmer voltage, 45 V; fragmentor voltage, 110 V. The mass spectrometer was operated in MS (50–1,100 *m/z*) and auto MS/MS (50–800 *m/z*) modes for the structural analysis of all compounds. Post-acquisition data processing was performed using the Agilent Mass Hunter Qualitative Analysis software (B.08.00). The accurate mass data, isotopic patterns, ion source fragmentation, MS/MS fragmentation patterns, MS databases, filtering approaches (mass defect filtering, diagnostic fragment ion filtering, background subtraction filtering, neutral loss filtering), and bibliographic searches were employed for the tentative identification of compounds in ELE (15, 38).

### 2.6 Antibacterial activity of ethanolic extract of nasturtium leaves

The reference strains *Escherichia coli* ATCC^®^ 25922, *Salmonella Typhimurium* ATCC^®^ 14028, and *Enterococcus faecalis* ATCC^®^ 29212 were obtained from the Food Microbiology Laboratory at Pontificia Universidad Javeriana. The strains were reactivated in Brain Heart Infusion (BHI) broth at 37 ± 2°C for 24 h at 110 RPM. Subsequently, they were isolated on BHI agar to confirm culture viability and purity (39).

#### 2.6.1 Samples preparation

Two samples of ELE were evaluated: one with myrosinase (ELE-Mi) and one without myrosinase (ELE-W). four grams of dry ELE were weighed and mixed with 8 g of phosphate buffer (pH 7) using a vortex mixer for 1 min. The solutions were then sonicated for 5 min and centrifuged for 10 min at 4,000 rpm. Finally, the extract was filtered using a 0.22 μm PVDF filter.

ELE-Mi was incubated with 7 μL of myrosinase (0.01 U/μL) per mL of extract for 15 min at 37°C 37 ± 2°C, and ELE-W was incubated with 7 μL of water under the same conditions (40).

#### 2.6.2 Determination of the minimum inhibitory concentration of ethanolic extract of nasturtium leaves

The Minimum Inhibitory Concentration (MIC) was determined using the broth microdilution method, following the guidelines of the Clinical and Laboratory Standards Institute (41). Serial two-fold dilutions of the nasturtium extract were prepared in Mueller-Hinton broth supplemented with 0.1% TTC (2,3,5-triphenyltetrazolium chloride), with concentrations ranging from 500 to 0.2 mg/mL. The plates were incubated at 37 ± 2°C for 24 h. Ampicillin (10 mg/mL) was used as a positive control, while broth without inoculum as a negative control. The bacterial strain concentration was adjusted to 10^8^ CFU/mL. The MIC was defined as the lowest extract concentration at which no growth or color change in TTC was observed, indicating complete inhibition. Each test was performed in quintuplicate (42).

### 2.7 Statistical analysis

The assays were performed in triplicate, and the results are presented as the mean ± standard deviation. Statistical analysis was performed using JASP software (JASP, Amsterdam, Netherlands). Mean comparisons were conducted using Tukey’s test at a 5% significance level. Additionally, Pearson correlation coefficients (r) were calculated to evaluate the relationship between bioactive compound content and antioxidant activity assays.

## 3 Results

### 3.1 Preliminary phytochemical characterization of nasturtium leaves and flowers

**Figure 1:**
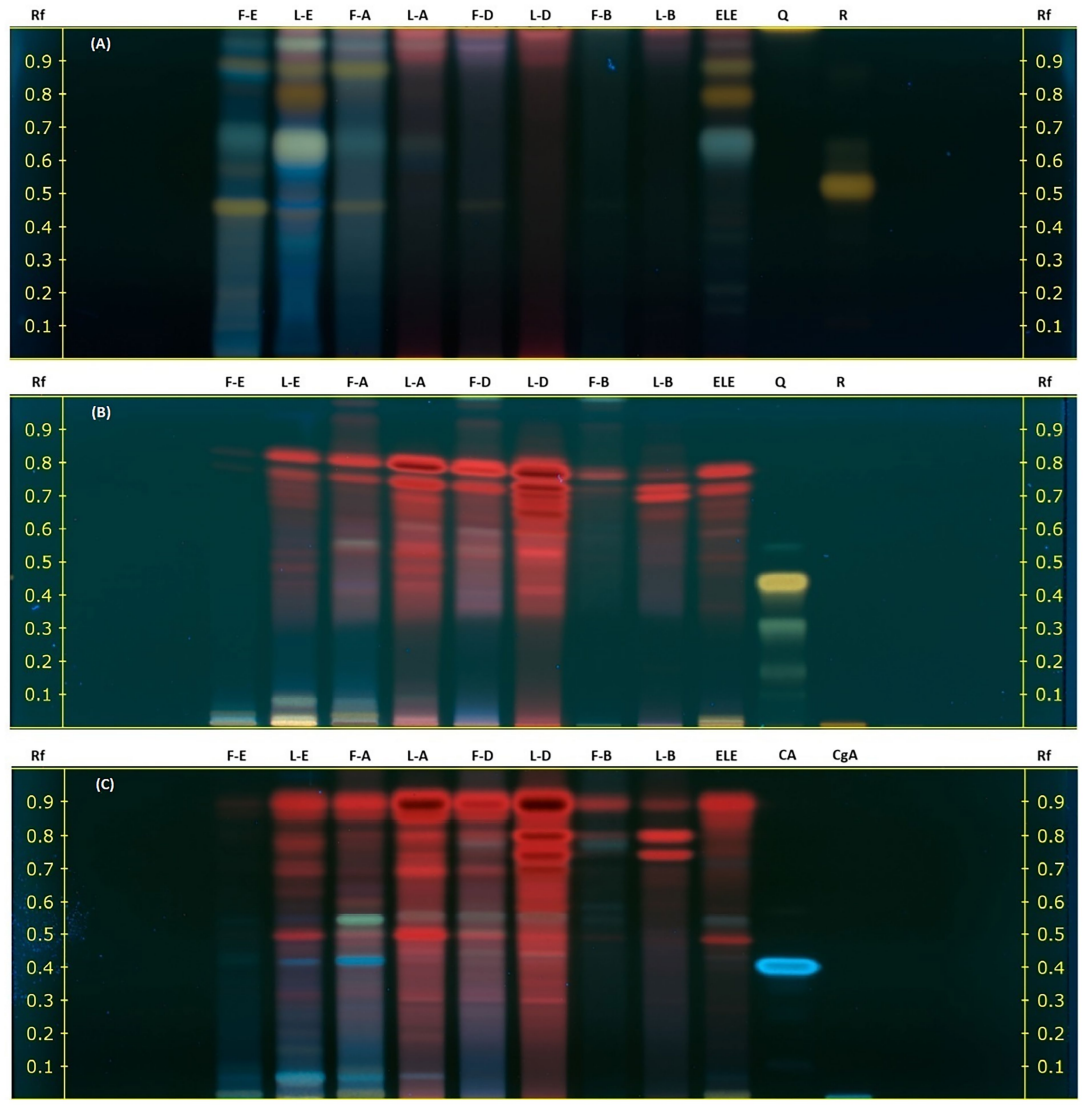
Preliminary phytochemical characterization of nine nasturtium leaf and flower extracts using HPTLC. Extracts: F, Flowers; L, Leaves; E, Ethanol; A, Ethyl Acetate; D, Dichloromethane; B, Benzine; and ELE, ethanolic extract for food. Standards: CA, Caffeic acid; CgA, Chlorogenic acid; Q, Quercetin; and R, Rutin. The chromatograms were developed using a natural reagent as a derivatizing agent and visualized under UV light at 366 nm. **(A)** Glycosylated flavonoids. **(B)** Aglycone flavonoids. **(C)** Phenolic derivatives.

The HPTLC analysis of ethanolic and ethyl acetate extracts from nasturtium leaves and flowers revealed that flavonoids and phenolic acids were the predominant compounds, as displayed in Figures 1A–C. Under the analytical conditions applied in this study, glycosylated flavonoids and their aglycones exhibited an intense orange-yellow fluorescence, as evidenced by the reference standard rutin (*R_f_* = 0.50) and quercetin (*R_f_* = 0.45) Figures 1A,B. Similarly, ethanol (F-E) and ethyl acetate (F-A) extracts from flowers, as well as ethanol extracts from leaves (L-E and ELE), displayed the distinctive yellow-orange fluorescence fingerprint of glycosylated flavonoids (*R_f_* 0.45–0.95) (43). Figure 1B displays the chromatographic plate used for the qualitative identification of flavonoid aglycones. Quercetin was included as a reference standard, yielding a distinctive yellow band at an *R_f_* = 0.45, which was not observed in the nasturtium extracts. Although no flavonoids aglycones were clearly detected by HPTLC, the presence of glycosyl flavonoids in nasturtium has been previously reported. These compounds are observed by us in Figure 1A. The orange-yellow bands were observed at *R_f_* values of 0.4–0.9, which indicates the presence of glycosylated flavonoids with a quercetin and kaempferol nucleus.

Meanwhile, the blue band observed in Figure 1C reveals the presence of phenolic acids in the L-E, F-A, and ELE extracts with *R_f_* values ranging from 0 to 0.50 and 0.40, corresponding to caffeic acid as a reference standard.

### 3.2 Characterization of nasturtium methanolic (MF and ML) and ethanolic (ELE) extracts: bioactive compounds and antioxidant capacity

#### 3.2.1 Qualitative analysis and quantification of GLSs

**Figure 2:**
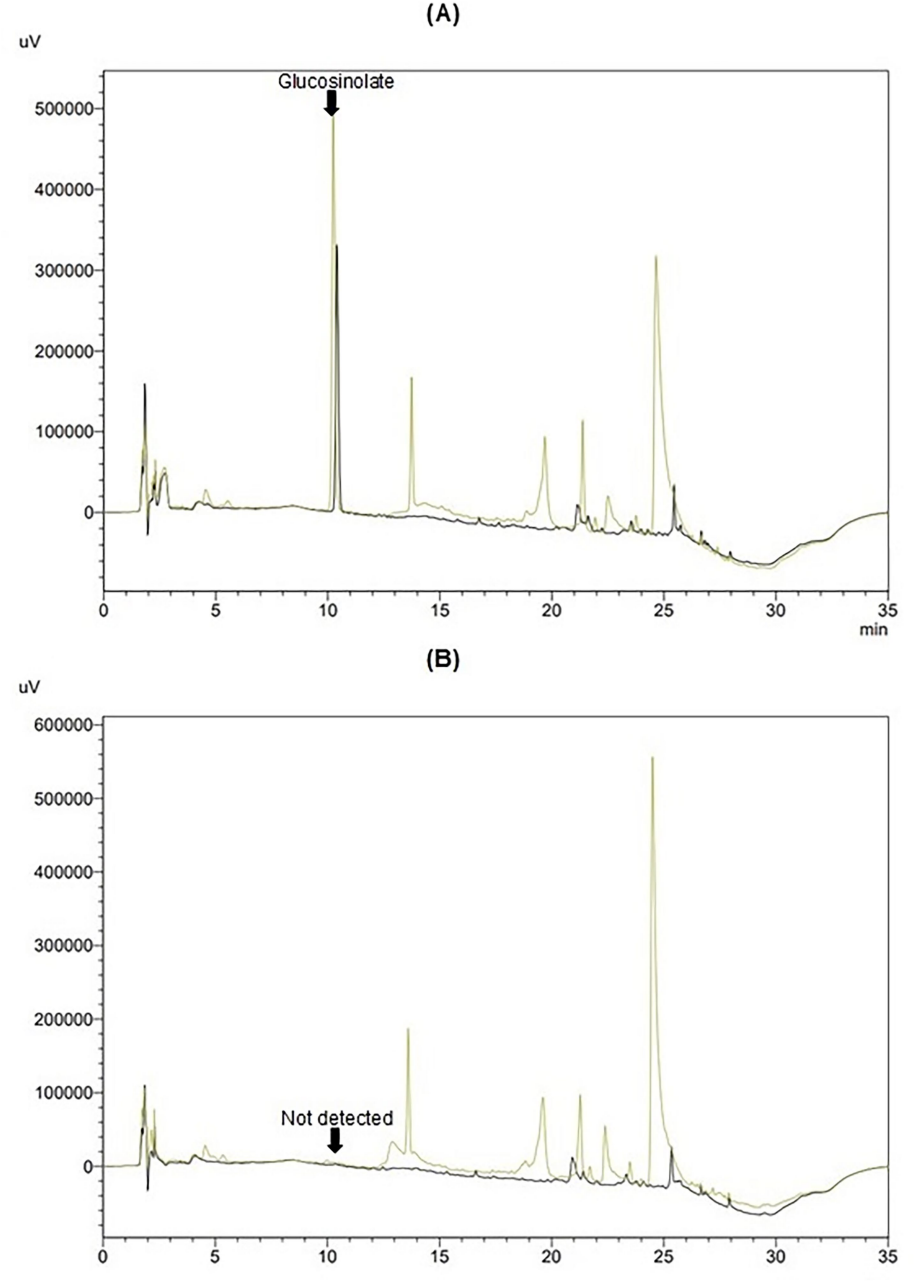
Chromatograms of methanolic extracts from flowers (MF, yellow) and leaves (ML, black): **(A)** without myrosinase and **(B)** after hydrolysis with myrosinase.

The qualitative identification of GLSs was achieved by incubating MF, ML, and ELE extracts with myrosinase, a process that eliminates the GLSs signal, as evidenced by MF and ML in Figures 2A,B. This process allows for the detection of GLSs based on their retention time.

The quantification of intact GLSs in MF, ML, and ELE was conducted using sinigrin as an external standard. Data was adjusted based on an average recovery of 96%, obtained by adding a surrogate standard to each sample analyzed. The results, presented in Table 2 show the highest GLSs content in MF (27.49) followed by ML (18.27) and ELE (8.47), expressed as μmol SE/g DS (*p <* 0.05). Different units to express content GLSs (mg SE/g DS and μg SE/g DS) were used for comparison purposes.

**Table 2:** Intact GLSs content in methanolic flowers (MF), methanolic leaves (ML), and ethanolic leaves (ELE) extracts.

Extract	Intact GLSs content in methanolic extracts			
	This work average ± standard deviation	Ceslová et al. (24)	Griffiths et al. (47)	Schreiner et al. (9)
ML	18.27^b^ ± 2.93 μmol SE/g DS 7.48 mg SE/g DS* 7,480 μg SE/g DS*	331 μg/g	0.63 μmol/g	3.13 mg/g
MF	27.49 ^c^ ± 2.30 μmol SE /DS 11.25 mg SE/g DS* 11,250 μg SE/g DS*	302–480 μg/g	–	6.36 mg/g
ELE	8.47 ^a^ ± 1.68 μmol SE/g DS 3.46 mg SE/g DS* 3,460 μg SE/g DS*			
LOD	0.53 μM			
LOQ	1.77 μM			
Equation	*y* = 15580147.93x-37064 (*R*^2^ = 0.9938)			

*Intact GLSs content was calculated in different units for comparison purposes. LOD and LOQ refer to the limits of detection and quantification, respectively. *R*^2^ represents the coefficient of determination. Statistically significant differences are indicated by letters (a–c), where (a) corresponds to the lowest value, followed by (b) and (c). SD: Standard deviation; DS: Dry sample.

The LOD and LOQ values were both below 2 μM, indicating that the method is suitable for detecting and quantifying glucosinolates in samples with low analyte concentrations. The method demonstrated high linearity, with a coefficient of determination (R^2^) greater than 0.99 the working concentration range. Accuracy, expressed as percent recovery, was calculated using sinigrin without extraction as the reference value (100% recovery). The extraction process resulted in a 9% decrease in recovery, indicating its impact on analytical quantification. This loss was accounted for by applying a correction to the results.

Precision analysis revealed a coefficient of variation (CV) of 4.6% at the lowest concentration tested (0.0005 mM), and less than 2.0% at higher concentrations in experimental replicates. For technical replicates, the CV was below 1.0%.

These results confirm that the chromatographic method developed is precise, accurate, and linear across the tested concentration range, making it suitable for the quantification of glucosinolates.

#### 3.2.2 Quantification of total phenolic and flavonoid content

**Figure 3:**
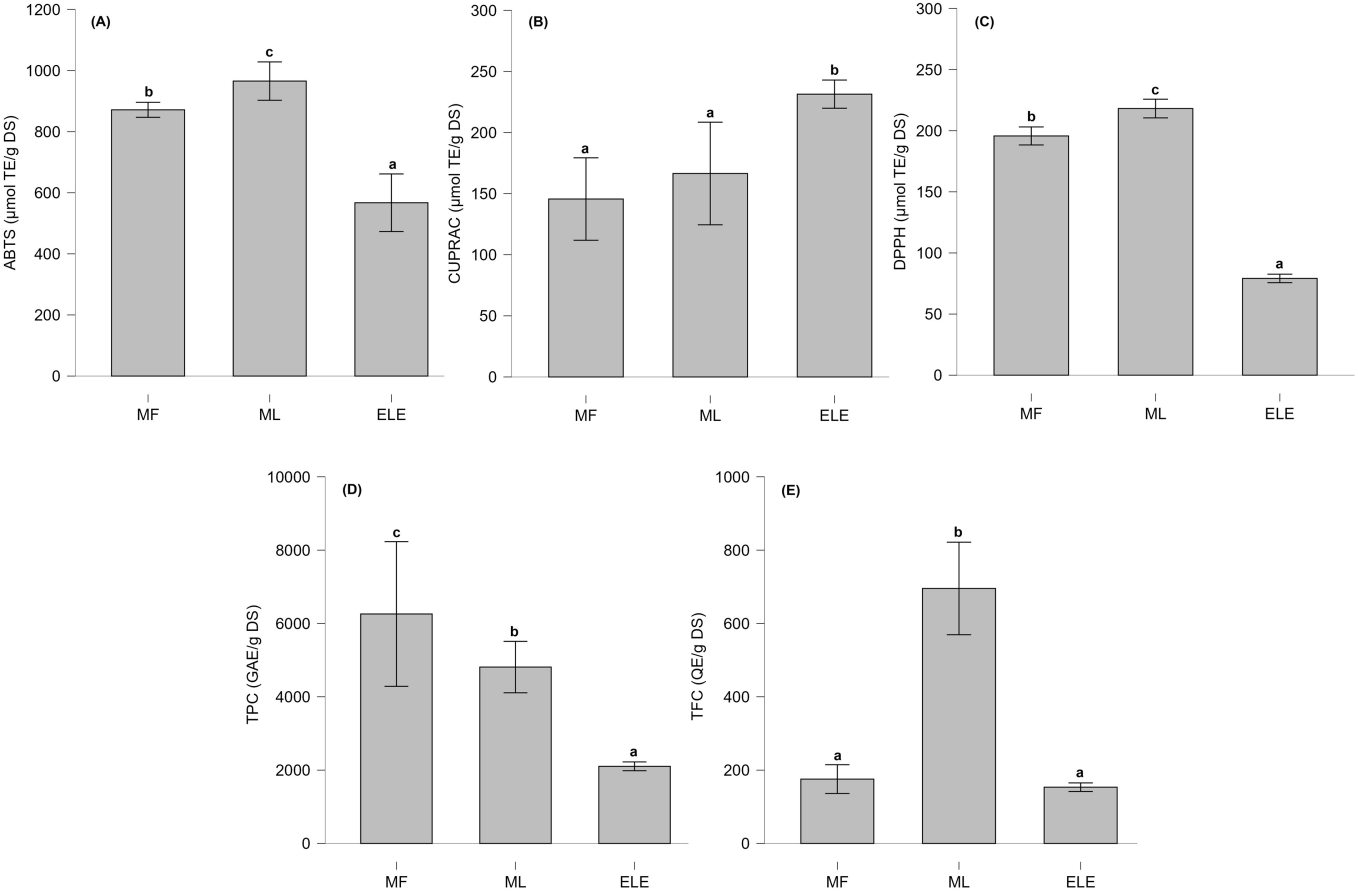
**(A–C)** Antioxidant capacity evaluated using different methodologies. **(D,E)** Total content of phenolic content (TPC) and total flavonoid content (TFC). Statistically significant differences are indicated by letters (a–c), where (a) represents the lowest value, followed by (b) and (c).

The total phenolic content (TPC) from MF, ML, and ELE in Figure 3D was 4.81 ± 0.28, 6.26 ± 0.79, and 2.10 ± 0.19 g GAE/g DS, respectively. All values were significantly different (*p* < 0.01), with MF exhibiting the highest TPC, followed by ML and ELE. Regarding the total flavonoid content (TFC), the values for MF, ML, and ELE in Figure 3E were 0.70 ± 0.05, 0.18 ± 0.02, and 0.15 ± 0.02 g QE/g DS, respectively. ML showed the highest content, and no significant difference was observed between MF and ELE (*p* > 0.05).

#### 3.2.3 Determination of antioxidant capacity of nasturtium extracts

The antioxidant capacity of MF, ML, and ELE, as assessed using the ABTS, CUPRAC and DPPH methods is present in Figures 3A–C. The antioxidant capacity values for ABTS, CUPRAC, and DPPH assays ranged from 526 to 994, 133 to 248, and 78 to 220 μmol TE/g DS, respectively.

**Figure 4:**
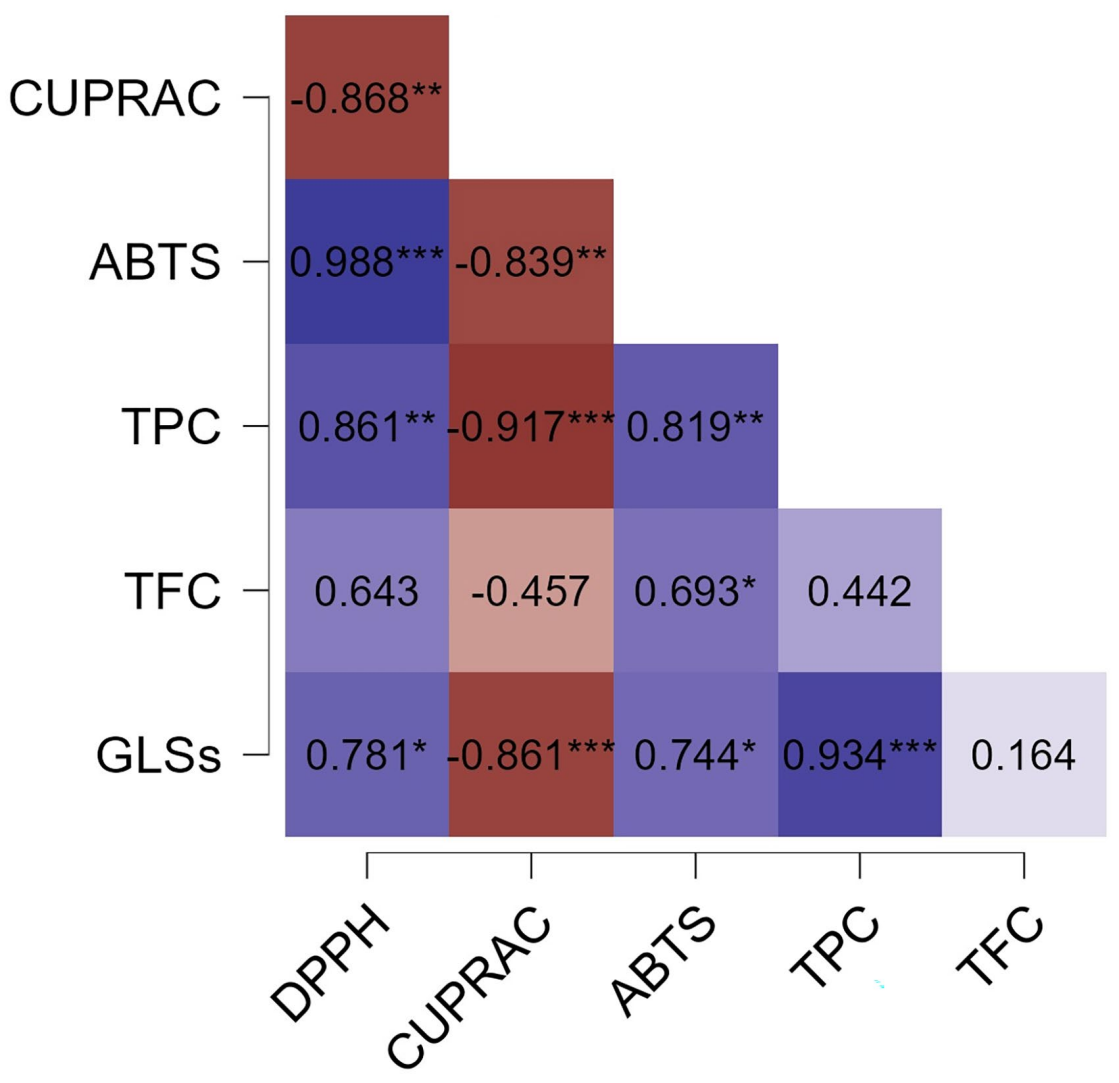
Pearson correlation heatmap showing the relationships between antioxidant capacity (DPPH, ABTS and CUPRAC expressed as μmol TE/g DS) of methanolic extracts (MF and ML) and ethanolic extract (ELE), as well as total phenolic content (TPC, expressed as g GAE/g DS), total flavonoid content (TFC, expressed as g QE/g DS), and glucosinolates (GLSs, expressed as μmol SE/g DS). Statistical significance levels are indicated as follows: no statistically significant differences (NS) (*p* > 0.05); * (*p* ≤ 0.05); ** (*p* ≤ 0.01); *** (*p* ≤ 0.001). DS, Dry sample.

Figure 4 shows the results of Pearson’s correlation analysis among all evaluated characteristics in the MF, ML, and ELE extracts. A significant positive correlation (*p* < 0.05) was observed between DPPH, ABTS, TPC and GLSs.

### 3.3 Analysis of the ethanolic extract by UHPLC-q-TOF-MS/MS

To further elucidate its chemical composition, a tentative identification of 15 compounds was carried out using UHPLC-q-TOF-MS/MS analysis.

The identification process was based on the analysis of [M – H]^−^ pseudo-molecular ions, isotopic patterns, and the fragmentation behavior of each signal. The detected compounds included a monosaccharide (compound **1**), a sulfur-containing compound (compound **3**), phenolic acids (compounds **4**, **5**, **6**, **7**, and **8**), flavonoids (compounds **10**, **12**, **13**, **14**, and **15**), and unidentified compounds (NI **2**, **9**, and **11**). The base peak chromatogram (BPC) is presented in Supplementary Figure 1. Compound **1,** tentatively identified as glucose, exhibited a deprotonated molecular ion at *m/z* 179.0562 [C_6_H_11_O_6_]^−^ and an MS/MS fragment at *m/z* 161.0464, corresponding to the loss of a water molecule. Compound **3** was assigned to benzyl glucosinolate.

Furthermore, the dissociation of [M – H]^−^ produces abundant ions and side chain fragments containing structural information, facilitating the assignment of benzyl glucosinolate to *m/z* 408.0424 in ELE.

**Figure 5:**
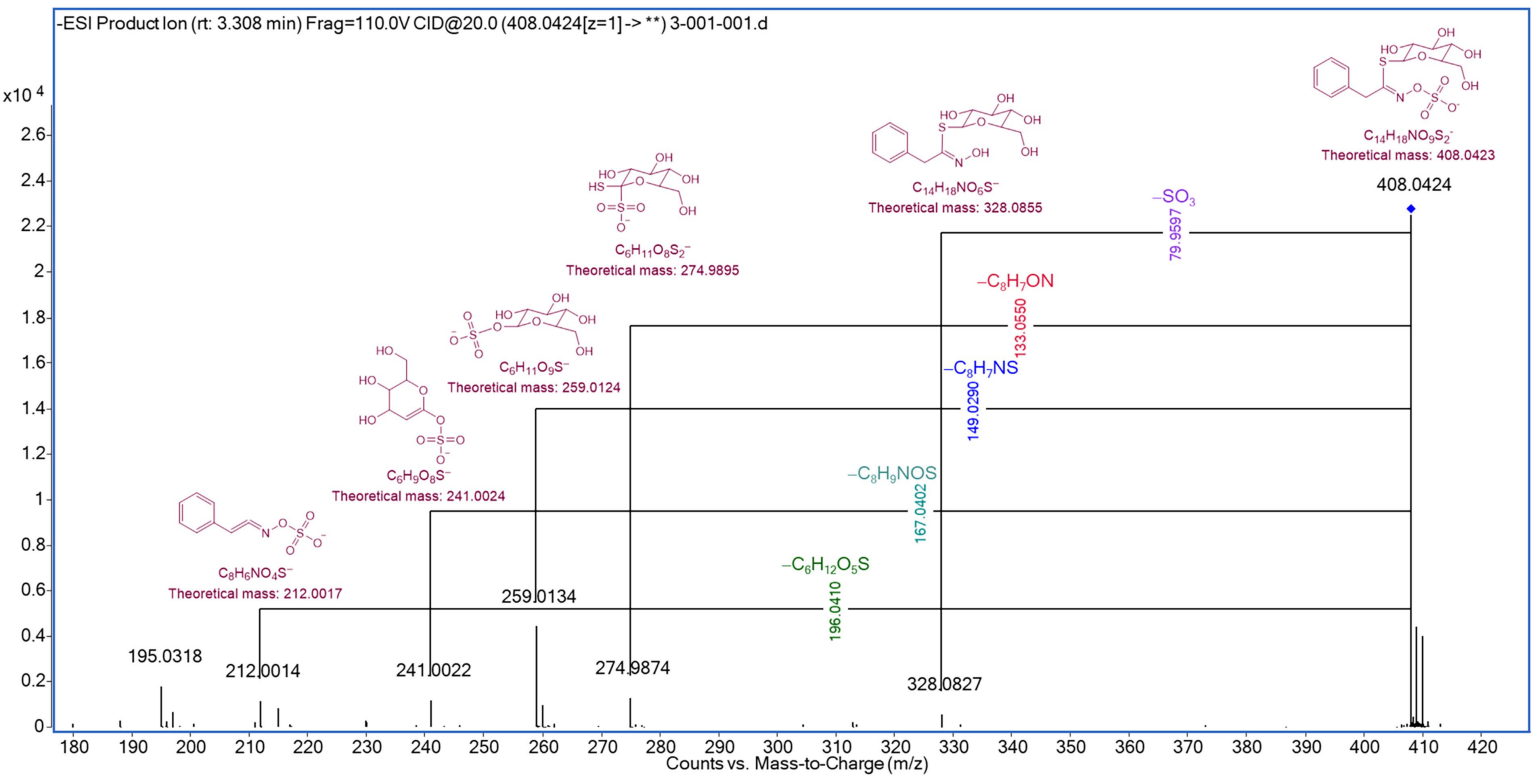
Detection of intact benzyl glucosinolate by UHPLC-q-TOF-MS/MS analysis.

The full-scan mass spectra of benzyl glucosinolate, along with the suggested structures, mass values, and formulas of the lost fragments leading to ion formation, are depicted in Figure 5.

The loss of specific fragments was observed for the ion m/z 328.0844, corresponding to the elimination of the sulfonate group from the intact glucosinolate, as described by Campos et al. (44), and for the ion m/z 274.9895, corresponding to the cleavage of the hydroximoyl moiety, as reported by Rollin et al. (45). Additionally, the ions *m/z* 259.0124, 241.0024, and 212.0014 were generated through fragmentation, followed by intramolecular rearrangements (45, 46).

Five phenolic acids derived from quinic acid (compounds **4**–**8**), were tentatively identified. Compounds **4** and **5** were assigned to isomers of caffeoylquinic acid, exhibiting *m/z* 353.0871 and 353.0879, respectively, with a deprotonated molecular formula [C_16_H_17_O_9_]^−^. Meanwhile, compounds **6**, **7,** and **8** were identified as derivatives of coumaroylquinic acid, with *m/z* values of 337.0914, 337.0908, and 337.0916, respectively.

Four flavonoids previously reported in nasturtium leaves and flowers were tentatively identified in ELE. Compound **10** was assigned to isoquercitrin with a deprotonated *m/z* of 463.0873 and a molecular formula [C_21_H_19_O_12_]^−^. Likewise, compounds **12**, **13**, **14**, and **15** were identified as flavonoids derived from quercetin and kaempferol, with *m/z* values of 505.0985, 447.0927, 533.0927, and 489.1037, respectively. Finally, compounds **2**, **9,** and **11** remained unidentified (Table 3).

**Table 3:** Tentative identification of 15 compounds in hydroethanolic extract of nasturtium leaves by LC-HRMS, including one monosaccharide (compound 1), sulfur-containing compounds (compounds 3), phenolic acids (compounds 4–8), flavonoids (compounds 10, 12–15), and unidentified compounds (NI 9, 2, and 11).

Compound	*R*_t_ (min)	[M-H]^−^ (*m/z*)	MS^2^ product ions (*m/z*)	Error (ppm)	Formula	Tentative identification	References
1	0.594	179.056	161.0464	0.49	C_6_H_12_O_6_	Glucose	
2	1.999	212.075	140.9905; 134.0478	1.07	C_10_H_15_NO_2_S	NI	
3	3.308	408.042	212.0017; 241.0024; 259.0124; 274.9895; 328.0855	0.25	C_14_H_18_NO_9_S_2_^−^	Glucotropaeolin	(71)
4	3.637	353.087	191.0572; 179.0504; 173.0459	−2.00	C_16_H_18_O_9_	Caffeoylquinic acid isomer (3-*O*-caffeoylquinic acid)	(72)
5	4.100	353.087	191.0565 179.0610	0.27	C_16_H_18_O_9_	Caffeoylquinic acid isomer (5-*O*-caffeoylquinic acid)	(73)
6	4.297	337.091	191.0567; 173.0446; 163.0385	−4.42	C_16_H_18_O_8_	3-*p*-Coumaroylquinic acid	(16)
7	4.518	337.090	191.0533; 173.0606; 163.0359; 119.0482 93.0332	−6.20	C_16_H_18_O_8_	4-*p*-Coumaroylquinic acid	(74)
8	4.707	337.091	191.0549; 173.0451	−3.83	C_16_H_18_O_8_	5-*p*-Coumaroylquinic acid	(72)
9	5.482	565.192	339.1229; 207.0521	−0.82	C_27_H_34_O_13_	NI	
10	5.624	463.087	300.0278: 271.0231; 163.0415	−0.76	C_21_H_20_O_12_	Isoquercitrin	(66)
11	5.740	487.304	443.1168; 177.1316	−3.93	C_29_H_44_O_6_	NI	
12	5.989	505.098	463.0881; 300.0274; 212.0752	−0.52	C_23_H_22_O_13_	Quercetin-3-acetyl-glucoside	(24)
13	6.128	447.092	349.4503; 284.0330; 255.0314: 227.0374	−1.31	C_21_H_20_O_11_	Kaempferol 3-*O*-glucoside	(75)
14	6.572	533.092	489.1029;284.0330; 255.0300; 227.0348	−1.84	C_24_H_22_O_14_	Kaempferol 3-*O*- (6′′-malonylglucoside)	(75)
15	7.010	489.103	477.1594; 424.4347; 334.8254; 255.0308	−0.31	C_23_H_22_O_12_	Kaempferol-*O*-acetylhexoxide	(76)

### 3.4 Antibacterial activity of nasturtium leaf ethanolic extract

In the present study, the antimicrobial activity of ELE incubated with myrosinase was evaluated. The minimum inhibitory concentration (MIC) values determined for ELE against three microbial species were as follows: *Escherichia coli and Salmonella Typhimurium*, both at 31.3 mg/mL, and *Enterococcus faecalis* at 15.6 mg/mL. All three tested strains were inhibited by the positive control (ampicillin, 10 mg/mL), while the negative control exhibited bacterial growth, thereby validating the assay.

The lowest MIC value was recorded for *Enterococcus faecalis* (15.6 mg/mL), suggesting a higher susceptibility of this bacterium to the extract. In contrast, *Escherichia coli* and *Salmonella Typhimurium* displayed an MIC of 31.3 mg/mL, indicating a lower sensitivity to the active compounds. Notably, the control extract without the addition of myrosinase, showed no antibacterial activity against any of the tested microorganisms.

## 4 Discussion

This investigation aimed to identify the main bioactive compounds in nasturtium and evaluate their antioxidant and antimicrobial potential, considering the possibility of using the extracts in food applications. Preliminary phytochemical characterization facilitated the general identification of metabolite families previously reported in polar extracts of nasturtium, including flavonoids and PC. Phytochemical characterization data for nasturtium flowers in Colombia are limited, as described in previous studies (11, 16), and the composition and abundance of these bioactive compounds may vary depending on geographical location, environmental conditions, and other factors. The compounds identified in the preliminary phytochemical analysis are consistent with previous reports describing the presence of flavonoids and phenolic acids in polar extracts of *Tropaeolum majus* L. (16, 24). Subsequently, two extracts were prepared with methanol and ethanol; these solvents are commonly used to extract bioactive compounds of interest, with methanol and mixtures of methanol/water being the most used for the extraction of PC and GLSs.

The content of GLSs, TPC, and TFC was analyzed independently in flower extracts (MF) and leaf (ML and ELE) extracts. The higher glucosinolate content observed in MF compared to ML and ELE is consistent with previously reported findings (9, 24). Additionally, the glucosinolate concentrations in MF, ML, and ELE (Table 2) exceeded the previously reported values for methanolic extracts (9, 24, 47). This discrepancy could be attributed to the lack of myrosinase inactivation during the extraction process (24, 47) in those studies, which could have led to glucosinolate degradation. Furthermore, the prolonged derivatization process required for converting GLSs to desulfoglucosinolates using sulfatase may explain the lower glucosinolate content observed, a phenomenon also reported for methanolic extracts of *Moringa oleifera* leaves (9, 15).

The analysis of GLSs content in plants presents challenges due to the influence of multiple factors on the profile and concentration of these bioactive compounds. These factors include: (i) environmental conditions such as soil composition, climate, fertilizer application, and UV exposure (9, 48); (ii) drying methods, including oven-drying and freeze-drying (49); (iii) extraction strategies, such as the desulfoglucosinolate method versus intact GLSs analysis (15); and (iv) myrosinase inactivation. Myrosinase, an enzyme compartmentalized within intact plant cells, is released upon tissue disruption, triggering GLSs hydrolysis into isothiocyanates (ITCs) (12, 50). Therefore, ensuring myrosinase inactivation is an important step in the characterization of GLSs in nasturtium to prevent underestimation of their content.

In the present study, the plant material was harvested in a greenhouse under unmodified conditions, without interventions aimed at enhancing bioactive compound content. However, previous studies have reported particularly high glucosinolate concentrations, ranging from 40 to 130 μmol/g, achieved through the selection of high-yielding varieties and the optimization of cultivation conditions, especially via sulfur application (51).

As for the TPC in MF, ML, and ELE, the values differed significantly (*p* < 0.01), with MF exhibiting the highest TPC, followed by ML and ELE. These differences may be due to the specific distribution of PC in nasturtium, as certain flavonoids, such as myricetin and its derivatives, are predominantly found in the flowers (16, 24). Regarding the TFC, the methanolic leaf extract showed the highest content, as other studies have shown that, due to the polarity of flavonoids, methanol is an efficient solvent for their extraction (52). Since the composition and abundance of secondary metabolites can vary widely depending on geographical location, environmental conditions, and other agronomic factors, and considering the limited phytochemical data available for nasturtium flowers cultivated in Colombia (11, 16), the present findings offer new insights into the composition of leaves and flowers grown under local conditions.

Given the diverse range of antioxidant compounds documented in nasturtium, including those identified in Colombian nasturtium in this study, assessing its antioxidant capacity was deemed relevant. While total phenolics and flavonoids are widely recognized for their direct antioxidant properties (53–56), GLSs themselves do not exhibit direct antioxidant activity; their hydrolysis product, benzyl isothiocyanate (BITC), has been linked to the modulation of transcription factors involved in the phase II antioxidant response upon ingestion or cellular stimulation with BITC (50, 57). The evaluation of antioxidant capacity was achieved using three distinct methodologies: DPPH, ABTS, and CUPRAC, each employing different mechanisms to inhibit substrate oxidation. The DPPH and ABTS assays rely on both Hydrogen Atom Transfer (HAT) and Single Electron Transfer (SET) mechanisms (58), whereas CUPRAC assay is exclusively based on SET (59). The sensitivity of these methods to specific compounds depends on solvent properties and pH conditions (60). Specifically, the DPPH assay, when performed in organic solvents, primarily evaluates the antioxidant capacity of hydrophobic compounds. Conversely, the ABTS and CUPRAC assays assess the antioxidant capacity of both hydrophilic and lipophilic compounds present in a mixture (58, 60). However, ABTS values were higher than those of CUPRAC and DPPH, indicating a greater affinity for determining the antioxidant capacity of the compounds in the analyzed extracts.

In this study, ML exhibited a significantly higher antioxidant capacity than MF and ELE in the DPPH and ABTS assays (*p* < 0.01), consistent with previously reported findings (24). In contrast, CUPRAC results showed that ELE exhibited the highest antioxidant capacity, with no statistically significant difference observed between MF and ML. This could be attributed to the presence of flavonoid glycosides in ELE, which undergo hydrolysis to their corresponding aglycones, as previously described (61). In addition, the PC and ascorbic acid contents may contribute to the observed antioxidant activity (11, 16, 24, 25). Previous analyses conducted by our group revealed that nasturtium leaves from Colombia contain 31.43 mg/100 g of ascorbic acid (data not shown), while other studies reported 71.5 mg/100 g of ascorbic acid in Colombian nasturtium flowers (11).

Pearson correlation analysis of the evaluated features in the MF, ML, and ELE extracts showed a significant positive correlation (*p* < 0.05) between DPPH, ABTS, TPC and GLSs. The strong correlation between DPPH and ABTS assays with TPC aligns with prior reports (62) on plant matrices rich in PC. Furthermore, the positive correlation between GLSs and TPC content is consistent with previous studies reporting the simultaneous accumulation of PC and GLSs in *Brassica rapa* L. *ssp. pekinensis* and *Brassica oleracea* var. *gongylodes* under different light conditions. Therefore, this correlation may depend on external factors specific to the crop, which were not evaluated in the present study (63, 64). Since both DPPH and ABTS methods measure the same chemical property—namely, the ability of antioxidants to donate hydrogen atoms or electrons—a similar antioxidant response in both assays was expected. In this case, TPC content appears to be a key factor influencing the antioxidant capacity of the extracts, as assessed by the DPPH and ABTS assays. TFC also exhibited a positive correlation with TPC, DPPH, and ABTS; however, this correlation was statistically significant only in the ABTS assay. This may be attributed to the greater sensitivity of the ABTS method to the antioxidant compounds present in the extracts, as illustrated in Figure 3A, where Trolox equivalent (TE) values were up to four times higher than those obtained using the DPPH and CUPRAC methods.

Although GLSs and PC, due to their polarity, are efficiently extracted with methanol, this solvent is not suitable for food applications due to toxicity concerns (65). In this study, a polar extract was prepared from nasturtium leaves using GRAS solvents, which are more appropriate for the development of functional food ingredients. Leaves were selected over flowers for the preparation of ELE due to their greater abundance in the crop.

The phytochemical characterization of ELE by HPTLC, along with the quantification of its TPC, TFC, and GLSs, supports its potential as a promising extract rich in bioactive compounds. However, a deeper understanding of the specific compounds responsible for its antioxidant and antibacterial properties is essential. To address this, UHPLC-q-TOF-MS/MS analysis was performed. Five phenolic acids derived from quinic acid, including caffeoylquinic acid and coumaroylquinic acid derivatives, were tentatively identified. These phenolic acids have been previously identified in both leaves and flowers of nasturtium by other authors (16, 24). Four flavonoids previously reported in nasturtium leaves and flowers were tentatively identified in ELE, among which isoquercitrin is recognized as the most abundant flavonoid in nasturtium, known for its diuretic properties (66). Likewise, four compounds were identified as flavonoids derived from quercetin and kaempferol. These flavonoids have been previously detected in nasturtium leaves and flowers (16, 24).

This tentative identification of ELE composition aligns with preliminary phytochemical assays, which confirmed the presence of phenolic acids and flavonoids in the ethanolic extract. These findings are further supported by the quantification of TPC and TFC. Furthermore, the quantification and tentative identification of GLSs confirmed the retention of intact benzyl glucosinolate, which was achieved using the hydroethanolic extraction methodology. This process was assisted by ultrasound and, importantly, by the inactivation of myrosinase, an essential factor in glucosinolate preservation. Moreover, the presence of antioxidant compounds, such as phenolic acids and flavonoids commonly extracted with polar solvents such as water, methanol, ethanol, and their mixtures are consistent with the results obtained for the antioxidant activity of the extract (67).

Furthermore, ELE was found to be abundant in benzyl glucosinolate, a compound that, in the presence of myrosinase, undergoes hydrolysis to form BITC, which is recognized for its antimicrobial properties, among other bioactivities. In this study, the ethanolic extract of nasturtium leaves (ELE) incubated with myrosinase showed varying antibacterial effects against the tested microorganisms. *Enterococcus faecalis* (Gram-positive) showed the highest susceptibility, with the lowest minimum inhibitory concentration (MIC) of 15.6 mg/mL, while *Escherichia coli* and *Salmonella Typhimurium* (both Gram-negative), had MIC values of 31.3 mg/mL, suggesting a higher susceptibility of *Enterococcus faecalis* to the extract. The results indicate that the antibacterial activity of the nasturtium extract, primarily composed of benzyl glucosinolate and PC, depends on the enzymatic hydrolysis of benzyl glucosinolate into benzyl isothiocyanate (BITC) by myrosinase.

Studies on *Salmonella Typhimurium* have shown that BITC alters bacterial cell morphology, damages cell membranes, and leads to the release of intracellular material, suggesting that its mechanism of action involves membrane disruption. Additionally, BITC reduces both intracellular and extracellular ATP levels, indicating potential interference with bacterial metabolism, likely through membrane disruption. Furthermore, BITC has been found to downregulate the expression of virulence genes (*hil*A, *hil*C, and *hil*D), which play key roles in the pathogenicity of *Salmonella Typhimurium*, potentially reducing its ability to invade host cells (27).

Isothiocyanates are highly reactive compounds that exert their antibacterial effects through multiple mechanisms, including the induction of oxidative stress. This process leads to the formation of reactive oxygen species (ROS) within bacterial cells, which in turn cause damage to membrane lipids, proteins, and nucleic acids, ultimately compromising cell viability (68). In addition, isothiocyanates can interact directly with essential protein functional groups, such as thiol (-SH) groups, forming covalent adducts. This interaction has been shown to inhibit critical metabolic processes, including energy production pathways (69).

In Gram-positive bacteria such as *Enterococcus faecalis*, the cell wall, rich in peptidoglycan and teichoic acids, does not appear to act as an effective barrier against isothiocyanates. The ability of teichoic acids to retain cations and interact with lipophilic compounds may facilitate the accumulation of isothiocyanates on the cell surface, enhancing their antibacterial effect (70). This could explain the greater susceptibility of *Enterococcus faecalis* to the extract.

In contrast, Gram-negative bacteria, including *Escherichia coli* and *Salmonella Typhimurium*, possess an outer membrane abundant in lipopolysaccharides, which act as a physical barrier against antibacterial compounds. Although isothiocyanates can penetrate this membrane, their initial resistance may reduce their effectiveness, requiring higher concentrations to achieve complete bacterial growth inhibition.

These findings highlight the potential of nasturtium extract (ELE) as a broad-spectrum natural antibacterial, showing possible applications in controlling foodborne pathogens relevant to food safety. Its mechanism of action may involve oxidative stress induced by benzyl isothiocyanate, membrane destabilization, and other pathways that warrant further investigation.

ELE, prepared with GRAS solvents, is suitable for use in food systems, particularly those with high water activity, and may contribute to shelf-life extension by providing antioxidant and antibacterial protection. Its GRAS status and simple extraction process suggest good scalability for industrial applications. Additional studies are needed to evaluate the antioxidant and antimicrobial activity of the extract in real food matrices, as the food environment can influence its efficacy. Moreover, its stability under different processing conditions (e.g., pasteurization, pH variation, storage time) should be assessed to support its commercial application. Finally, *in vitro* and *in vivo* evaluations of ELE functionality are essential to advance our understanding of its potential as an ingredient in functional foods and nutraceuticals.

Furthermore, to support the use of ELE, further investigation into their functionality and applicability is necessary. Complementary studies should focus on evaluating their bioavailability and functional properties through *in vitro* and *in vivo* models, including protection and delivery systems for their bioactive compounds such as encapsulation techniques. These assays are essential for deepening our understanding of their potential and advancing their implementation as ingredients in functional foods and nutraceuticals.

## Data Availability

The original contributions presented in the study are included in the article/Supplementary material, further inquiries can be directed to the corresponding author.
