# Correction: Chemical profiling, antioxidant, and antibacterial properties of *Tropaeolum majus* L. extract as a functional food ingredient

**DOI:** 10.3389/fnut.2025.1714061

**Published:** 2025-11-13

**Authors:** Eliana-Yissel Aguilera-Angel, Diego Ballesteros-Vivas, Ricardo Vera-Bravo, Néstor García, Jorge-Eliecer Robles-Camargo, Geison Modesti Costa, Mauricio Espinal-Ruiz, Juan Pablo Caicedo-Trejos, Ana Karina Carrascal Camacho, Izlia-Jazheel Arroyo-Maya, Elena Ibáñez, Alejandro Cifuentes, Valentina Guzmán-Pérez

**Affiliations:** 1 Departamento de Nutrición y Bioquímica, Facultad de Ciencias - Pontificia Universidad Javeriana, Bogotá, Colombia; 2 Escuela de Ciencias Básicas Tecnología e Ingeniería, Grupo DAVINCI, Universidad Nacional Abierta y a Distancia, Bogotá, Colombia; 3 Departamento de Química, Facultad de Ciencias - Pontificia Universidad Javeriana, Bogotá, Colombia; 4 Departamento de Biología, Facultad de Ciencias - Pontificia Universidad Javeriana, Bogotá, Colombia; 5 Center for Research in Energy and Environment (CREE), University of Missouri of Science and Technology, Rolla, MO, United States; 6 Laboratorio de Microbiología de Alimentos, Grupo de Biotecnología Ambiental e Industrial, Departamento de Microbiología, Facultad de Ciencias - Pontificia Universidad Javeriana, Bogotá, Colombia; 7 Departamento de Procesos y Tecnología - División de Ciencias Naturales e Ingeniería, Universidad Autónoma Metropolitana – Unidad Cuajimalpa, Ciudad de México, Mexico; 8 Foodomics Laboratory, Institute of Food Science Research (CIAL) (CSIC-UAM), Madrid, Spain

**Keywords:** nasturtium, *Tropaeolum majus*, benzyl glucosinolate, antioxidant capacity, antibacterial activity

Author Néstor García was erroneously spelled as Néstor-Julio García-Castro.

The author contributions statement was erroneously given as

“E-YA-A: Conceptualization, Investigation, Methodology, Writing – original draft, Writing – review & editing, Formal analysis, Software, Validation. DB-V: Conceptualization, Formal analysis, Investigation, Methodology, Software, Validation, Writing – original draft, Writing – review & editing, Supervision. RV-B: Formal analysis, Investigation, Methodology, Supervision, Writing – original draft, Writing – review & editing. N-JG-C: Writing – original draft, Writing – review & editing, Funding acquisition, Resources. J-ER-C: Writing – original draft, Writing – review & editing, Investigation, Methodology, Supervision. GC: Methodology, Writing – original draft, Writing – review & editing, Formal analysis. ME-R: Formal analysis, Methodology, Writing – original draft, Writing – review & editing, Conceptualization. JC-T: Methodology, Writing – original draft, Writing – review & editing. AKCC: Formal analysis, Investigation, Methodology, Writing – original draft, Writing – review & editing, Funding acquisition, Resources. I-JA-M: Formal analysis, Investigation, Methodology, Writing – original draft, Writing – review & editing. EI: Methodology, Writing – original draft, Writing – review & editing. AC: Methodology, Writing – original draft, Writing – review & editing, Investigation. VG-P: Investigation, Methodology, Writing – original draft, Writing – review & editing, Conceptualization, Funding acquisition, Project administration, Supervision.”

The correct author contributions statement is

“E-YA-A: Conceptualization, Investigation, Methodology, Writing – original draft, Writing – review & editing, Formal analysis, Software, Validation. DB-V: Conceptualization, Formal analysis, Investigation, Methodology, Software, Validation, Writing – original draft, Writing – review & editing, Supervision. RV-B: Formal analysis, Investigation, Methodology, Supervision, Writing – original draft, Writing – review & editing. NG: Writing – original draft, Writing – review & editing, Funding acquisition, Resources. J-ER-C: Writing – original draft, Writing – review & editing, Investigation, Methodology, Supervision. GC: Methodology, Writing – original draft, Writing – review & editing, Formal analysis. ME-R: Formal analysis, Methodology, Writing – original draft, Writing – review & editing, Conceptualization. JC-T: Methodology, Writing – original draft, Writing – review & editing. ACa: Formal analysis, Investigation, Methodology, Writing – original draft, Writing – review & editing, Funding acquisition, Resources. I-JA-M: Formal analysis, Investigation, Methodology, Writing – original draft, Writing – review & editing. EI: Methodology, Writing – original draft, Writing – review & editing. ACi: Methodology, Writing – original draft, Writing – review & editing, Investigation. VG-P: Investigation, Methodology, Writing – original draft, Writing – review & editing, Conceptualization, Funding acquisition, Project administration, Supervision.”

There was a mistake in Table 2, page 9 as published. There were errors in the bibliographic references cited in Table 2.

The corrected Table 2 appears below.

**Table 2:** Intact GLSs content in methanolic flowers (MF), methanolic leaves (ML), and ethanolic leaves (ELE) extracts.

**Extract**	**Intact GLSs content in methanolic extracts**			
	**This work average** ±**standard deviation**	**Česlová et al. (24)**	**Griffiths et al. (47)**	**Schreiner et al. (9)**
ML	• 18.27^b^ ± 2.93 μmol SE/g DS • 7.48 mg SE/g DS^*^ • 7,480 μg SE/g DS^*^	331 μg/g	0.63 μmol/g	3.13 mg/g
MF	• 27.49^c^ ± 2.30 μmol SE/DS • 11.25 mg SE/g DS^*^ • 11,250 μg SE/g DS^*^	302–480 μg/g	–	6.36 mg/g
ELE	• 8.47^a^ ± 1.68 μmol SE/g DS 3.46 mg SE/g DS^*^ 3,460 μg SE/g DS^*^			
LOD	0.53 μM			
LOQ	1.77 μM			
Equation	*y* = 15,580,147.93*x* – 37,064 (*R*^2^ = 0.9938)			

^*^Intact GLSs content was calculated in different units for comparison purposes. LOD and LOQ refer to the limits of detection and quantification, respectively. *R*^2^ represents the coefficient of determination. Statistically significant differences are indicated by letters (a–c), where (a) corresponds to the lowest value, followed by (b) and (c). SD, standard deviation; DS, dry sample.

The original version of this article has been updated.

