# Supplementary material for: Chemical profiling, antioxidant, and antibacterial properties of Tropaeolum majus L. extract as a functional food ingredient
Source: Front Nutr. 2025 Sep 24;12:1626562. doi: 10.3389/fnut.2025.1626562 (PMC12507335; doi:10.3389/fnut.2025.1626562)
Supplement: Supplementary file 1 [file Data_Sheet_1.PDF]

Supplementary Material

Supplementary Figures 1- 2

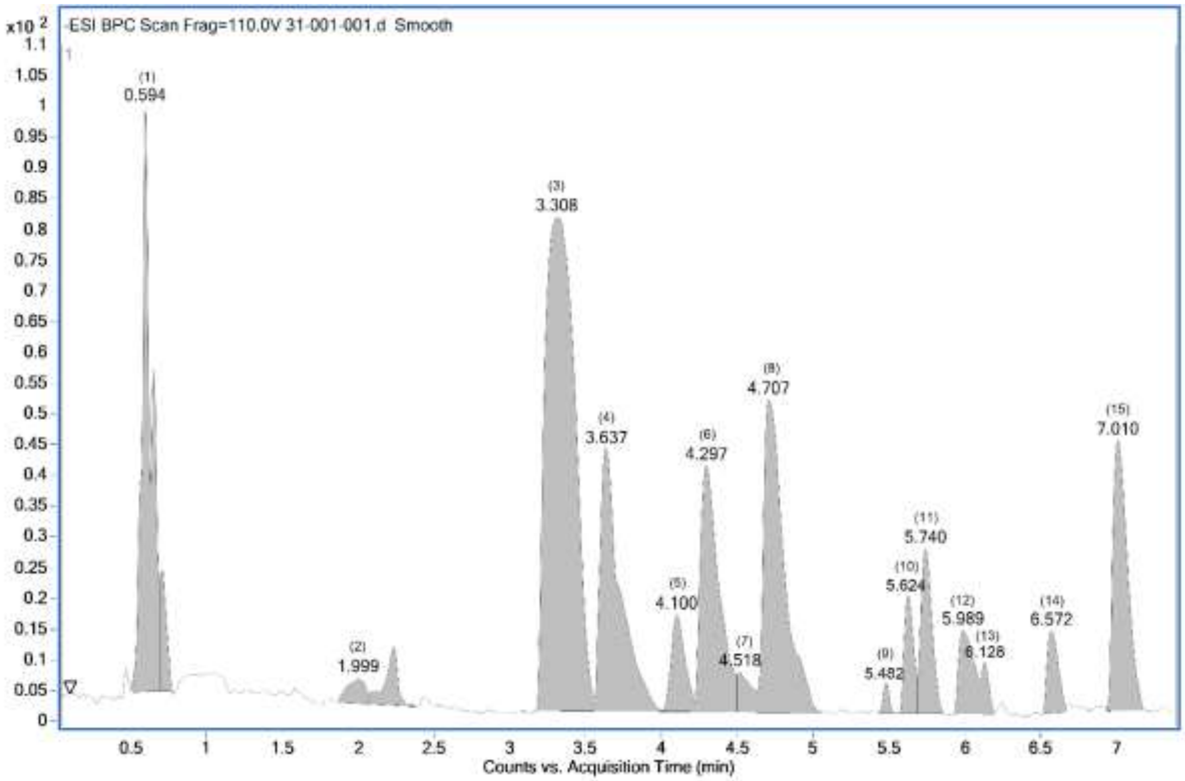

Supplementary Figure 1. Base peak chromatogram (BPC) of ethanolic extract from leaves (ELE). The numbers in parentheses correspond to the identification of the 15 compounds described in Table 3, obtained by UHPLC-q-TOF-MS/MS analysis.
